# Epigenetic alteration and microRNA dysregulation in cancer

**DOI:** 10.3389/fgene.2013.00258

**Published:** 2013-12-03

**Authors:** Hiromu Suzuki, Reo Maruyama, Eiichiro Yamamoto, Masahiro Kai

**Affiliations:** Department of Molecular Biology, Sapporo Medical UniversitySapporo, Japan

**Keywords:** microRNA, tumor suppressor, oncomir, CpG island methylation, histone modification, biomarker, EZH2

## Abstract

MicroRNAs (miRNAs) play pivotal roles in numerous biological processes, and their dysregulation is a common feature of human cancer. Thanks to recent advances in the analysis of the cancer epigenome, we now know that epigenetic alterations, including aberrant DNA methylation and histone modifications, are major causes of miRNA dysregulation in cancer. Moreover, the list of miRNA genes silenced in association with CpG island hypermethylation is rapidly growing, and various oncogenic miRNAs are now known to be upregulated via DNA hypomethylation. Histone modifications also play important roles in the dysregulation of miRNAs, and histone deacetylation and gain of repressive histone marks are strongly associated with miRNA gene silencing. Conversely, miRNA dysregulation is causally related to epigenetic alterations in cancer. Thus aberrant methylation of miRNA genes is a potentially useful biomarker for detecting cancer and predicting its outcome. Given that many of the silenced miRNAs appear to act as tumor suppressors through the targeting of oncogenes, re-expression of the miRNAs could be an effective approach to cancer therapy, and unraveling the relationship between epigenetic alteration and miRNA dysregulation may lead to the discovery of new therapeutic targets.

## INTRODUCTION

MicroRNAs (miRNAs) are endogenous, small, non-coding single-stranded RNAs about 22 nucleotides in length, which function at the post-transcriptional level as negative regulators of gene expression (He and Hannon, 2004). Each miRNA negatively regulates its target genes in one of two ways, depending on the degree of complementarity between itself and its target messenger RNAs (mRNAs). miRNAs that bind to mRNA sequences with perfect or nearly perfect complementarity induce the RNA-mediated interference (RNAi) pathway, in which mRNA transcripts are cleaved by a miRNA-associated RNA-induced silencing complex (miRISC). This mechanism is mainly observed in plants, though miRNA-directed mRNA cleavage does occur in animals. Most animal miRNAs are thought to act by binding to imperfectly complementary sites within the 3′ untranslated regions (UTRs) of target mRNAs, thus inhibiting the initiation of translation via the miRISC.

Annotation of their genomic locations suggests that many miRNA genes are located within intergenic regions, though they are also found within exonic and intronic regions in either the sense or antisense orientation. Like genes encoding proteins, miRNA genes are mainly transcribed by RNA polymerase II. They are initially transcribed as large precursors, called primary miRNAs (pri-miRNAs), which may encode multiple miRNAs in a polycistronic arrangement. Pri-miRNAs are then processed by the RNase III enzyme Drosha and its cofactor DGCR8/Pasha to produce ~70-nt hairpin-structured second precursors, called pre-miRNAs. The pre-miRNAs are transported to the cytoplasm by the nuclear export protein Exportin-5 (XPO5), after which they are processed by another RNase III enzyme, DICER, to generate mature miRNA products.

Sequences of miRNAs are highly conserved among species, and play critical roles in a variety of biological processes, including cell proliferation, development, differentiation, and apoptosis. In addition, subsets of miRNAs are thought to act as tumor suppressor genes or oncogenes, and their dysregulation is a common feature of human cancers (Esquela-Kerscher and Slack, 2006; Croce, 2009). More specifically, expression of miRNAs is generally downregulated in tumor tissues, as compared to corresponding healthy tissues, which suggests some miRNAs behave as tumor suppressors in some tumors. Although the mechanism underlying the alteration of miRNA expression in cancer is still not fully understood, recent studies have shown that cancer affects multiple mechanisms involved in regulating miRNA levels. For example, a significant number of miRNAs are located within cancer-associated genomic regions or in fragile sites (Calin et al., 2004). The first report of altered miRNA expression in cancer was related to the frequent chromosomal deletion and downregulated expression of miR-15 and miR-16, two miRNAs thought to target the antiapoptotic factor B cell lymphoma 2 (BCL2) in chronic lymphocytic leukemia (CLL; Calin et al., 2002). More recent studies indicate that genetic mutations affecting proteins involved in the processing and maturation of miRNA, such as TARBP2 and XPO5, can also lead to overall reductions in miRNA expression (Melo et al., 2009, 2010). In addition, epigenetic alterations, including aberrant DNA methylation and histone modifications, appear to be a major mechanism by which the normal patterns of miRNA expression are disrupted in cancer. In this review, we will highlight the contribution made by epigenetic alteration of miRNAs to cancer, and discuss their clinical application as biomarkers and therapeutic targets.

## IDENTIFICATION OF EPIGENETICALLY DYSREGULATED miRNAs IN CANCER

The first evidence that epigenetic mechanisms are involved in silencing miRNAs in cancer came from a pharmacological unmasking experiment. Using a miRNA microarray, Saito et al. (2006) analyzed the expression profiles of miRNAs in T24 human bladder cancer cells and LD419 human normal fibroblasts treated with or without the DNA methyltransferase (DNMT) inhibitor 5-aza-2′-deoxycytidine (5-aza-dC) and the histone deacetylase (HDAC) inhibitor 4-phenylbutyric acid (4-PBA). Among the genes upregulated in the cancer cells was miR-127, which was embedded within a CpG island and was upregulated in association with DNA demethylation, acetylation of histone H3 and trimethylation of histone H3 lysine 4 (H3K4me3), which are marks of active transcription. Experimental evidence confirmed that the proto-oncogene BCL6 is a target of miR-127, suggesting miR-127 acts as a tumor suppressor (Saito et al., 2006).

**FIGURE 1:**
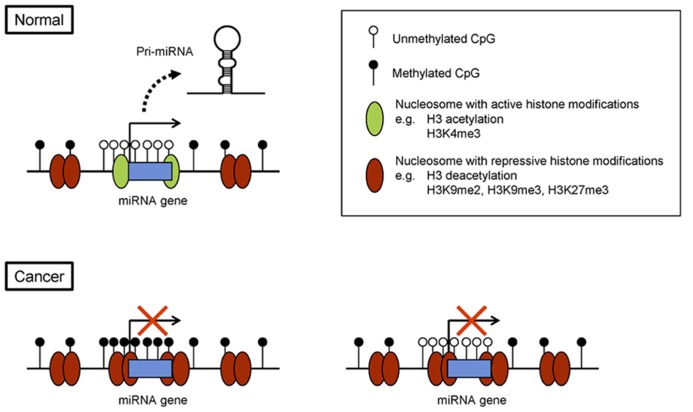
**Epigenetic silencing of miRNA genes in cancer.** Example of a miRNA gene with its CpG island. In normal cells, the CpG island is unmethylated, and the chromatin is associated with active histone modifications. In cancer cells, CpG island hypermethylation and repressive histone marks lead to epigenetic silencing of the miRNA gene (left). Alternatively, some miRNA genes are silenced through repressive histone modifications, rather than DNA methylation (right).

As with protein-coding genes, epigenetic regulation of miRNA genes is tightly associated with histone modification (**Figure 1**). As mentioned, H3K4me3 and acetylation of histone H3 lysine 9/14 are hallmarks of active miRNA gene promoters in embryonic stem cells and in cancer cells (Marson et al., 2008; Ozsolak et al., 2008; Suzuki et al., 2011). By contrast, di- or trimethylation of histone H3 lysine 9 (H3K9me2 or H3K9me3) and trimethylation of lysine 27 (H3K27me3) are marks of repression. For instance, a combination of chromatin immunoprecipitation (ChIP)-on-chip and miRNA microarray analyses in prostate cancer cells revealed that miRNA expression correlates positively with H3K4me3 and correlates inversely with H3K27me3 in miRNA promoter regions (Ke et al., 2009). In addition, genome-wide screening for miRNA genes with reduced levels of H3K4me3 and increased levels of H3K9me2 led to the identification of 13 miRNA genes, including the miR-124 family, miR-9 family, and miR-34b/c, that are epigenetically silenced in acute lymphoblastic leukemia (ALL; Roman-Gomez et al., 2009). To assess genome-wide histone modifications, we recently performed deep sequencing (ChIP-seq) in colorectal cancer (CRC) cells and identified the putative promoter regions of 174 pri-miRNA genes (Suzuki et al., 2011). By searching for miRNAs that showed upregulated expression and increases in H3K4me3 marks upon DNA demethylation, we identified 37 miRNA genes as potential targets of epigenetic silencing in CRC cells.

Epigenetically silenced miRNA genes were also identified through genome-wide DNA methylation analysis. For instance, methylation microarray analysis using the Infinium BeadChip revealed miR-10b to be a target of DNA methylation in gastric cancer (GC; Kim et al., 2011). In addition, Yan et al. (2011) performed a genome-wide methylome analysis entailing deep sequencing of MBD (methylated DNA binding domain)-isolated DNA in HCT116 cells, and identified a number of methylated genes, including miR-941, miR-1237, and miR-1247. And Baer et al. (2012) carried out an integrative analysis of genome-wide DNA methylation and histone modification (H3K4me3) in CLL and identified 128 miRNAs that carried aberrant DNA methylation at their promoters. Interestingly, of those 128 miRNA promoters, 38 exhibited hypermethylation, while 90 showed hypomethylation, which are indicative of epigenetically silenced and activated miRNAs, respectively. In fact, the hypermethylated regions included a number of well-defined epigenetically silenced miRNA genes, including miR-9-2, miR-124-2, and miR-129-2, while the hypomethylation was accompanied by upregulation of several miRNAs, including miR-21, miR-34a, and miR-155.

## ABERRANT DNA METHYLATION OF miRNA GENES IN CANCER

Among the rapidly growing list of miRNAs that are now known to be aberrantly methylated in cancer, many are downregulated in association with CpG island hypermethylation, while some are upregulated via hypomethylation of their CpG island. Here we describe well-characterized miRNA genes showing aberrant DNA methylation in cancer.

Epigenetic silencing of miR-124 family genes was first discovered in CRC (Lujambio et al., 2007), and they are now known to be methylated in several other types of malignancy, including ALL (Agirre et al., 2009), non-Hodgkin’s lymphoma (Wong et al., 2011a), and liver (Furuta et al., 2010), pancreatic (Wang et al., 2013), renal (Gebauer et al., 2013), and cervical cancer (Wilting et al., 2010). Within the human genome, three independent loci (miR-124-1, miR-124-2, and miR-124-3) encode identical mature forms of miR-124, and all are associated with CpG islands, which may be targets of hypermethylation in cancer (Lujambio et al., 2007). miR-124 exerts its tumor suppressor function by targeting cyclin-dependent kinase 6 (CDK6), and epigenetic silencing of miR-124 reportedly results in CDK6 activation and Rb phosphorylation (Lujambio et al., 2007; Agirre et al., 2009). In ALL, miR-124 methylation is associated with higher recurrence and mortality rates, and may be an independent prognostic factor for disease-free and overall survival (Agirre et al., 2009). miR-124 family genes are also frequently methylated in the gastric mucosa of *Helicobacter pylori*-positive healthy individuals, suggesting their methylation could be induced by chronic inflammation (Ando et al., 2009).

Members of the miR-34 gene family (miR-34a, miR-34b, and miR-34c) are direct targets of p53, and their ectopic expression in cancer cells induces cell cycle arrest and apoptosis (Bommer et al., 2007; He et al., 2007). Within the human genome, miR-34a is located on chromosome 1p36, while miR-34b and miR-34c are co-transcribed from a single transcription unit on chromosome 11q23. The promoters of both genes are targets of CpG island hypermethylation in multiple malignancies including oral (Kozaki et al., 2008), esophageal (Chen et al., 2012), gastric (Suzuki et al., 2010), colorectal (Toyota et al., 2008), lung (Wang et al., 2011b), breast and renal cancer (Lodygin et al., 2008; Vogt et al., 2011), and hematological malignancies (Roman-Gomez et al., 2009; Wong et al., 2011b). Methylation of miR-34b/c has also been linked to cancer metastasis (Lujambio et al., 2008) and invasion (Watanabe et al., 2012). In addition, methylation-associated silencing of miR-34c was recently shown to promote self-renewal and epithelial–mesenchymal transition (EMT) in breast tumor-initiating cells (Yu et al., 2012). These findings, as well as their contribution to the p53 network, strongly imply that miR-34 family members act as tumor suppressors in cancer. Introduction of miR-34b/c into cancer cells leads to the downregulation of candidate target genes, including MET, CDK4, cyclin E2 (CCNE2), and MYC (Lujambio et al., 2008; Toyota et al., 2008). Likewise, restoration of endogenous miRNA expression through demethylation also downregulates target genes, suggesting miRNAs could be important targets for epigenetic cancer therapy (Toyota et al., 2008).

The CpG islands of miR-9 family genes (miR-9-1, miR-9-2, and miR-9-3) are also frequently methylated in various types of malignancies, including ALL (Roman-Gomez et al., 2009) and colorectal (Bandres et al., 2009), breast (Lehmann et al., 2008), pancreatic (Omura et al., 2008), and GCs (Tsai et al., 2011). Moreover, a screen for miRNA gene methylation in metastatic cancer cell lines identified miR-9 family genes as being methylated (Lujambio et al., 2008). Consistent with that finding, methylation of miR-9-1 is reportedly associated with lymph node metastasis in CRC (Bandres et al., 2009), while methylation of miR-9-1 and miR-9-3 correlates with metastatic recurrence of renal cell carcinoma (Hildebrandt et al., 2010). miR-9 has been shown to target fibroblast growth factor receptor 1 (FGFR1) and CDK6 in ALL (Rodriguez-Otero et al., 2011) and caudal-type homeobox 2 (CDX2) in GC (Rotkrua et al., 2011), suggesting a tumor-suppressive function.

The miR-200 gene family (miR-200a, miR-200b, miR-200c, miR-141, and miR-429) and miR-205 encode key regulators of EMT that act by directly targeting zinc finger E-box binding homeobox 1 (ZEB1) and ZEB2, which are transcriptional repressors that downregulate E-cadherin (CDH1; Gregory et al., 2008; Korpal et al., 2008; Park et al., 2008). Within the human genome, miR-200 family genes are grouped into two polycistronic units, miR-200b/200a/429 and miR-200c/141, located on chromosomes 1 and 12, respectively (Davalos et al., 2012). In normal mammary epithelial cells and fibroblasts, expression of miR-200 family and miR-205 genes is regulated by DNA methylation, histone modifications, or a combination of the two (Vrba et al., 2010), but aberrant DNA methylation leads to the silencing of these miRNAs in cancer (Ceppi et al., 2010; Neves et al., 2010; Wiklund et al., 2011). For instance, methylation of miR-200c/141 is tightly correlated with the invasive capacity of breast cancer cells (Neves et al., 2010). Similarly, in non-small cell lung cancer, promoter methylation is associated with loss of miR-200c expression, which is in turn associated with poor differentiation, lymph node metastasis, and weaker E-cadherin expression (Ceppi et al., 2010). Davalos et al. (2012) demonstrated that the upstream CpG islands of both units (miR-200b/200a/429 and miR-200c/141) are unmethylated in cancer cells with epithelial features, but are both methylated and silenced in transformed cells with mesenchymal characteristics.

In addition to its therapeutic implications, miRNA gene methylation could be a useful molecular marker for detecting cancer and/or predicting its outcome. For instance, the CpG island of miR-34b/c is methylated in more than 90% of primary CRCs, and methylation was detected in 75% of fecal specimens from CRC patients and in 16% of specimens from high-grade dysplasia patients, suggesting miR-34b/c methylation could be a useful feces-based screening marker (Kalimutho et al., 2011). It was also recently shown that miR-34a methylation and high levels of c-MET and β-catenin expression may be powerful predictive markers of liver metastasis in CRC (Siemens et al., 2013). In addition, miR-34b/c methylation was found to be elevated in the background gastric mucosa of multiple GC patients (Suzuki et al., 2010), and a subsequent study revealed that miR-34b/c methylation could be a marker for predicting the risk of metachronous GC (Suzuki et al., 2013). miRNA gene methylation is also detectable in urine specimens, and could be a useful marker of urinary tract cancer. A recent screening for epigenetically silenced miRNAs in bladder cancer cells identified methylation of four miRNA genes (miR-137, miR-124-2, miR-124-3, and miR-9-3), and their methylation in urinary DNA was found to be a useful biomarker of bladder cancer (Shimizu et al., 2013).

Many miRNA genes are reportedly downregulated in association with DNA hypermethylation in cancer, but some are epigenetically activated via DNA hypomethylation. As mentioned, a recent comprehensive analysis of miRNA in CLL revealed that approximately 60% of aberrantly methylated miRNA genes exhibited hypomethylation (Baer et al., 2012). For instance, the CpG island of let-7a-3 is heavily methylated in normal cells but is hypomethylated in lung adenocarcinoma, leading to its elevated expression (Brueckner et al., 2007). In lung cancer cells, let-7a-3 exerts oncogenic effects through actions on several genes involved in cell proliferation, adhesion, and differentiation. In addition, miR-200a and miR-200b are overexpressed in pancreatic cancer due to their hypomethylation, and their elevation in the serum of pancreatic cancer patients means they could potentially serve as diagnostic biomarkers (Li et al., 2010). miR-196 family genes (miR-196a and miR-196b) are located within the HOX gene cluster and are often overexpressed in tumors, which is indicative of their oncogenic functions (Luthra et al., 2008; Maru et al., 2009; Popovic et al., 2009; Guan et al., 2010). miR-196b is embedded within a CpG island, and its overexpression in GC is associated with its hypomethylation (Tsai et al., 2010).

Recently, Fornari et al. (2012) reported that miR-519d is upregulated due to DNA hypomethylation in hepatocellular carcinoma (HCC). miR-519d belongs to the chromosome 19 miRNA cluster (C19MC), which is the largest miRNA cluster in the human genome. miRNAs in the C19MC are normally expressed specifically in placenta (Bentwich et al., 2005), but DNA demethylation leads to their re-expression in cancer cells, which is indicative of their epigenetic repression in healthy tissue (Tsai et al., 2009; Suzuki et al., 2011). Upregulation of miR-519d, which is observed in approximately 50% of HCCs, is positively associated with CpG island hypomethylation and wild-type p53 (Fornari et al., 2012). miR-519d is thought to act as an oncogenic miRNA (oncomir) through its targeting of p21, PTEN, AKT3, and TIMP2.

## miRNA DYSREGULATION CAUSES ABERRANT DNA METHYLATION

**FIGURE 2:**
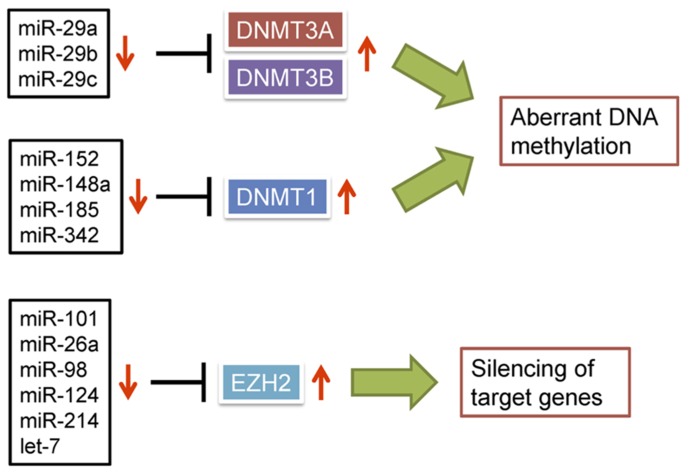
**Dysregulation of miRNAs is causally related to epigenetic alterations in cancer.** miRNAs able to negatively regulate DNMTs or EZH2 are frequently downregulated in cancer, which leads to epigenetic silencing of target genes.

Several lines of evidence support the idea that dysregulation of miRNAs can lead to aberrant DNA methylation in cancer. For instance, the miR-29 family (miR-29a, miR-29b, and miR-29c), which is downregulated in lung cancer, directly targets DNMT3A and DNMT3B (Fabbri et al., 2007; **Figure 2**). Ectopic expression of the miR-29 family in lung cancer cells restores expression of methylation-silenced tumor suppressor genes, including fragile histidine triad (FHIT) and WW domain containing oxidoreductase (WWOX). In addition, miR-143 is frequently downregulated in CRC cells, where it normally targets DNMT3A (Ng et al., 2009), and downregulated expression of miR-152 in HBV-related HCC correlates with increased expression of DNMT1 (Huang et al., 2010). Forced expression of miR-152 in liver cell lines reduces DNMT1 expression and global DNA methylation, whereas inhibition of miR-152 causes global DNA hypermethylation and increased methylation of the glutathione *S*-transferase pi 1 (GSTP1) and CDH1 promoter regions. Similarly, DNMT1 is targeted by miR-148a and miR-152 in cholangiocarcinoma cells, and their ectopic expression suppresses DNMT1 and induces expression of the tumor suppressor genes Ras association domain family 1A (RASSF1A) and p16 (Braconi et al., 2010). miR-342 was found to be downregulated in CRC cells, and restoration of its expression downregulated DNMT1 and reactivated expression of cancer-related genes through demethylation of their promoter regions (Wang et al., 2011a). miR-185 is downregulated in glioma cells in association with loss of heterozygosity (LOH), and its restoration reduces global DNA methylation and leads to re-expression of hypermethylated genes through targeting DNMT1 (Zhang et al., 2011b).

miR-34b was recently shown to target both DNMTs and HDACs in prostate cancer cells (Majid et al., 2013). As in other malignancies, miR-34b is silenced in association with CpG island methylation in prostate cancer, and low miR-34b expression is strongly associated with poor survival. Interestingly, ectopic expression of miR-34b in prostate cancer cells suppressed DNMTs and HDACs and induced partial demethylation and active chromatin modification of the endogenous miR-34b gene, which suggests a positive feedback loop. Collectively, these results indicate that dysregulation of specific miRNAs may be causally related to aberrant methylation of promoter CpG islands.

## HISTONE MODIFICATIONS AND miRNA DYSREGULATION

It is now evident that histone modifications also play a major role in the dysregulation of miRNAs in cancer. For example, treating a breast cancer cell line with the HDAC inhibitor LAQ824 induced upregulation or downregulation of a number of miRNAs within as little 5 h (Scott et al., 2006). Not only does this suggest the involvement of epigenetic mechanisms in the regulation of miRNAs in cancer cells, it also highlights the importance of secondary effects driven by miRNAs induced or downregulated through drug treatment. In addition, recent studies have shown that HDAC silences tumor-suppressive miRNAs in cancer. It is well documented that loss of miR-15a and miR-16 in CLL is associated with 13q loss; however, these miRNAs are also often downregulated in CLL samples without observable deletions in 13q, and Sampath et al. (2012) found that overexpression of HDACs (HDAC1, HDAC2, and HDAC3) is associated with downregulation of miR-15a, miR-16, and miR-29b. Furthermore, inhibition of the HDACs induced robust accumulation of active histone marks at the promoters of the miRNAs and increased their expression, which in turn led to downregulation of their target genes, BCL2 and MCL1. In another study, MYC interacted with HDAC3, which then colocalized to the promoters of miR-15a/miR-16-1 and their host gene DLEU2, resulting in MYC-induced suppression of these miRNAs in mantle cell lymphoma (Zhang et al., 2012a). And in HCC, upregulation of HDACs (HDAC1–3) was associated with repression of miR-449, which led to activation of the putative miR-449 target gene c-MET (Buurman et al., 2012).

In other settings, histone acetylation is involved in the activation of oncomirs in cancer. For example, miR-224 is commonly upregulated in HCC, and there is reportedly a positive correlation between miR-224 expression and histone acetylase protein EP300 in HCC tumors (Wang et al., 2012). It is well documented that the breast cancer susceptibility gene BRCA1 is involved in DNA damage repair and cell cycle regulation, but a recent study revealed an interesting link between BRCA1 and the epigenetic regulation of oncomirs. Chang et al. (2011) showed that wild-type BRCA1 epigenetically represses miR-155 by recruiting HDAC2 to the miR-155 promoter, while a BRCA1 R1699Q mutant relieves the repression and causes miR-155 to be overexpressed.

As mentioned, miRNA gene transcription is closely associated with histone modifications; thus some miRNA genes are silenced without DNA hypermethylation in cancer cells (**Figure 1**). For example, downregulation of miR-212 in lung cancer cells is reportedly associated with H3K9me2 and H3K27me3 but not DNA hypermethylation (Incoronato et al., 2011; **Figure 1**). miR-212 exerts a pro-apoptotic effect in lung cancer cells by targeting the anti-apoptotic gene PED, and inhibition of HDAC and the histone methyltransferase EZH2 strongly reactivates miR-212 expression in lung cancer cells. It was also recently found that miR-708 is repressed by H3K27me3 in metastatic breast cancer (Ryu et al., 2013). miR-708 targets neuronatin (NNAT), a regulator of intracellular Ca^2^^+^, and silencing miR-708 leads to elevation of intracellular Ca^2^^+^ levels and increased cell migration and metastasis.

## miRNA DYSREGULATION CAUSES ABERRANT HISTONE MODIFICATIONS

Dysregulation of miRNAs can also lead to aberrant histone modifications. EZH2 is a member of the polycomb group (PcG) of proteins, which are key regulators that silence numerous developmental genes (Schuettengruber et al., 2007). EZH2 functions as a catalytic subunit of polycomb repressive complex 2 (PRC2), which trimethylates H3K27. The available evidence suggests that EZH2 has oncogenic properties, and its overexpression in prostate and breast cancers promotes tumorigenesis, invasiveness and metastasis (Varambally et al., 2002; Kleer et al., 2003). Varambally et al. (2008) reported that EZH2 is a target of miR-101, and genomic loss of miR-101 is an important cause of EZH2 overexpression in cancer. Reduced expression of miR-101 and upregulation of EZH2 occur in parallel during the progression of prostate cancer, and genomic loss of miR-101 is more frequently seen in metastatic disease than localized cancers. Moreover, the loss of miR-101 and resultant overexpression of EZH2 appears to alter the global chromatin structure in cancer (Friedman et al., 2009). The inverse association between miR-101 and EZH2 has now been seen in bladder, gastric, lung, and renal cancer (Friedman et al., 2009; Wang et al., 2010; Zhang et al., 2011a; Sakurai et al., 2012). In addition, several other miRNAs, including miR-26a (Wong and Tellam, 2008), miR-98 (Alajez et al., 2010), miR-124 (Zheng et al., 2012), miR-144 (Guo et al., 2013), miR-214 (Derfoul et al., 2011), and let-7 (Kong et al., 2012) are also reported to negatively regulate EZH2 (**Figure 2**). Thus, dysregulation of miRNAs appears to be one of the major causes of EZH2 overexpression in cancer.

Overexpression of EZH2 also leads to the silencing of multiple miRNAs in cancer. It was recently demonstrated that EZH2 is frequently upregulated in primary HCCs, and miRNA expression profiling in HCC cells with EZH2-knockdown revealed that a set of miRNAs, including miR-139-5p, miR-125b, let-7c, miR-101, and miR-200b, are epigenetically suppressed by EZH2 in HCC (Au et al., 2012). Interestingly, miR-200b reportedly targets another PRC2 subunit, SUZ12, in breast cancer stem cells (Iliopoulos et al., 2010), suggesting a possible feedback loop between EZH2 overexpression and miRNA silencing in cancer. In another study, Cao et al. (2011) demonstrated that in prostate and breast cancer cell lines, EZH2 represses a set of miRNAs (miR-181c, miR-181b, miR-200b, miR-200c, and miR-203), which in turn negatively regulate the PRC1 subcomponents BMI1 and RING2. The inverse correlation between miRNA and PRC protein levels were further confirmed in prostate cancer tissues. These results are indicative of an integral regulatory axis involving PRC1, PRC2, and the epigenetic silencing of miRNAs in cancer.

Another recent study demonstrated the involvement of EZH2 and HDAC3 in MYC-mediated miRNA repression. In aggressive B cell lymphoma, miR-29a is repressed by MYC within a co-repressor complex that also includes HDAC3 and EZH2 (Zhang et al., 2012b). Interestingly, MYC contributes to EZH2 upregulation through repression of miR-26a, which targets EZH2, while EZH2 upregulates MYC by inhibiting miR-494, which targets MYC. It thus appears a positive MYC-miRNA-EZH2 feedback loop may mediate persistent overexpression of MYC and EZH2. Combined inhibition of HDAC3 and EZH2 induced restoration of miR-29 and suppressed lymphoma cell growth, suggesting the MYC–EZH2–miRNA axis could be a promising target for epigenetic therapy in B cell lymphoma.

## CONCLUDING REMARKS

In this review, we highlighted the relationship between epigenetic alteration of miRNAs and cancer. Aberrant DNA methylation commonly underlies miRNA dysregulation in cancer, and methylation of a subset of miRNA genes may be a useful biomarker for detecting cancer and/or predicting clinical outcome. Alteration of the histone modification pattern also leads to abnormal miRNA expression. In addition, recent findings suggest that miRNA dysregulation is causally related to aberrant DNA methylation and histone modifications that leads to genome-wide epigenetic abnormalities. It is anticipated that additional study of the relationship between epigenetic regulation and miRNAs will lead to the discovery of new biomarkers as well as therapeutic targets.

## Conflict of Interest Statement

The authors declare that the research was conducted in the absence of any commercial or financial relationships that could be construed as a potential conflict of interest.
